# P‐wave indices as predictors of atrial fibrillation

**DOI:** 10.1111/anec.12751

**Published:** 2020-04-10

**Authors:** Maria Uggen Rasmussen, Preman Kumarathurai, Andreas Fabricius‐Bjerre, Bjørn Strøier Larsen, Helena Domínguez, Ulla Davidsen, Thomas Alexander Gerds, Jørgen K. Kanters, Ahmad Sajadieh

**Affiliations:** ^1^ Department of Cardiology Copenhagen University Hospital of Bispebjerg Copenhagen Denmark; ^2^ Department of Biostatistics University of Copenhagen Copenhagen Denmark; ^3^ Laboratory of Experimental Cardiology Department of Biomedical Sciences University of Copenhagen Copenhagen Denmark

**Keywords:** atrial fibrillation, electrocardiography, P wave, P‐wave indices

## Abstract

**Background:**

P‐wave duration (P_DURATION_) and P‐wave area (P_AREA_) have been linked to risk of atrial fibrillation (AF), but they do not improve the efficacy of Framingham AF risk score. We suggest the incorporation of both variables in one index, the P‐wave area/P‐wave duration (P_AREA__/__DURATION_) index, which may be considered an expression of the average amplitude of the P wave that reflects aspects of P‐wave morphology.

**Objective:**

To assess the prognostic value of P‐wave area/P‐wave duration index (P_AREA/DURATION_ index) in lead II together with other P‐wave indices (PWIs) in incidence of AF in the Copenhagen Holter Study.

**Methods:**

The study included 632 men and women, between 55 and 75 years with no apparent heart disease or AF. Baseline standard 12‐lead Electrocardiography (ECGs) were analyzed manually.

**Results:**

The median follow‐up time was 14.7 (14.5;14.9) years. A total of 68 cases of AF and 233 cases of death were recorded. The restricted cubic spline method showed a U‐shaped association between P_AREA/DURATION_ and rate of AF. The lowest quintile of P_AREA/DURATION_ index in lead II was associated with increased rate of AF, HR 2.80 (1.64–4.79). The addition of the new index to the Framingham model for AF improved the model in this population. The P_AREA_ in lead II in its lowest quintile was also associated with increased rate of AF, HR 2.16 (1.25–3.75), but did not improve the Framingham model. P_DURATION_ and P‐wave terminal force (PTF) were not significantly associated with AF.

**Conclusion:**

A flat P wave as expressed by a small P_AREA/DURATION_ index in lead II is associated with increased rate of incident AF beyond known AF risk factors.

## 1 INTRODUCTION

Atrial fibrillation (AF) is the most common arrhythmia of clinical significance (Kirchhof et al., 2016). AF is known to increase cardiovascular and all‐cause mortality as well as risk of complications such as ischemic stroke, heart failure, and dementia (Kirchhof et al., 2016). Identifying patients at risk of developing AF is important in preventing AF‐related complications, such as stroke. The electrocardiogram may be a valuable tool in identifying these patients, as it is a cheap, noninvasive, and readily available method for evaluating electrophysiological function.

The P wave is a measure of atrial electrical activity. Previous studies have stipulated that P‐wave indices (PWI) may be useful in determining which patients are at risk of developing AF (Aizawa, 2017; German, Kabir, Dewland, Henrikson, & Tereshchenko, 2016; Magnani et al., 2015). Such measures include P‐wave duration (P_DURATION_), P‐wave area (P_AREA_), and P‐wave terminal force (PTF). PWIs are hypothesized to be surrogate measures of atrial electrical function and reflect subclinical atrial remodeling (Magnani, 2015). P_DURATION_ is generally accepted as a marker of atrial conduction time (Conte et al., 2017) and both increased P_DURATION_ and PTF are thought to be markers of left atrial enlargement (Shenasa, Josephson, & Mark Estes, 2015). P_AREA_ has been suggested as a marker of abnormal atrial structure (German et al., 2016). However, studies have shown varying results and a single, reliable marker for AF has not been identified.

**Figure 1:**
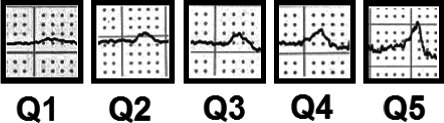
Examples of P‐wave shape in quintiles of P‐wave area/P‐wave duration index in lead II. The figure shows examples of P‐wave shape taken from the included electrocardiography s in this study, divided into 5 quintiles. The index is a representation of the average amplitude of the P wave. A small index (Q1) arises when the ratio between area and duration is skewed and the denominator is larger relative to the numerator, meaning a longer P‐wave duration and a smaller P‐wave area, hence, a flat, prolonged P wave. A large index (Q5) is a representation of a ratio with a small denominator and a large numerator, being a short, peaked P wave. The 3 middle quintiles represent normal P waves where the ratio between P‐wave area and P‐wave duration is not skewed

Abnormal P_AREA_ and P_DURATION_ may be considered to reflect different aspects of atrial structural and electrophysiological abnormalities (Conte et al., 2017; German et al., 2016; Shenasa et al., 2015). We suggest the P‐wave area/P‐wave duration index (P_AREA/DURATION_ index), as a new P‐wave index. This index can be considered a calculated expression of the average p‐wave amplitude, since it is the ratio between the area and the duration of the P wave. Integrating both P_AREA_ and P_DURATION_ in one single index that may reflect different electrophysiological abnormalities may provide a better measure of left atrial abnormalities and a better marker of AF. Furthermore, P_AREA/DURATION_ reflects some aspects of the P‐wave shape, where a small P_AREA/DURATION_ index may be considered a marker for a flat P wave and a large P_AREA/DURATION_ index as a marker of a peaked, short P wave (Figure 1). Recognizing these key morphological features makes it easier for clinicians to catch P‐wave abnormalities.

The objective of the study was to evaluate the association of P_AREA/DURATION_ index together with other PWI, including PTF, P_AREA_, P_DURATION,_ and incident AF in patients with no apparent heart disease at baseline. Furthermore, the study aimed to investigate the effect of adding PWI to the Framingham AF risk score (Schnabel et al., 2009).

## 2 METHODS

### 2.1 Copenhagen Holter Study

The Copenhagen Holter Study included middle‐aged and elderly subjects, with no apparent history of AF, stroke, or cardiovascular disease, enrolled between April 1998 and June 2000. Follow‐up was completed in 2014, with a median of approximately 14 years of follow‐up. The aim of the follow‐up study was to examine the value of 48‐hr Holter recording in assessing future adverse events including AF, ischemic stroke, and mortality in middle‐aged and older men and women. Information about the study protocol and the selection process has been published previously (Kumarathurai et al., 2017; Larsen, Kumarathurai, Falkenberg, Nielsen, & Sajadieh, 2015).

In short, all men of 55 years and all men and women of 60, 65, 70, and 75 years of age (*n* = 2,969) living within 2 defined postal regions in the city of Copenhagen were contacted with a questionnaire regarding cardiovascular risk factors, medication use, and medical history. Subjects were subsequently ranked according to several self‐reported risk factors including hypertension, diabetes mellitus, smoking habits, familial predisposition to sudden death or acute myocardial infarction, obesity (body mass index (BMI) > 30 kg/m^2^), or known hypercholesterolemia. All responding individuals with >1 risk factor, and 60% of subjects with 0–1 risk factors, selected at random, were invited to a follow‐up consisting of a physician‐based questionnaire, physical examination, laboratory testing, electrocardiography (ECG), and 48‐hr continuous ECG recording. Subjects with current or past AF, manifest ischemic heart disease, congestive heart failure, valvular heart disease, congenital heart disease, angina pectoris, stroke, cancer, or other life‐threatening conditions were excluded. This resulted in 678 participants who underwent fasting laboratory tests, a physical examination with anthropometric measurements, baseline ECG and up to 48 hr of continuous Holter monitoring. For the purpose of this study, out of the 678 participants, 648 had an ECG that was eligible for analysis. Of these, 16 participants were excluded because of missing variables, resulting in 632 subjects included in the analysis.

### 2.2 Follow‐up and endpoints

Data on death and AF were obtained from national registries. Discharge letters from hospital admissions and patient‐files were reviewed where necessary. Diagnosis of incident AF was verified with documentation in the form of ECG, telemetry or both from the patient records.

### 2.3 Ethics

The Copenhagen Holter Study was approved by the Regional Ethics Committee of Copenhagen and Frederiksberg. The study complies with the Helsinki Declaration. All participants provided their written informed consent.

### 2.4 ECG analysis

Baseline ECGs were analyzed manually, digitally using an image measurement program (ImageJ, Fiji) (Schindelin et al., 2012), divided between two raters and blinded for patient outcomes. The raters measured PTF in precordial lead V_1_ and P_DURATION_ and P_AREA_ in lead II. A predefined manual was used to ensure a standardized method of measuring by both raters.

**Figure 2:**
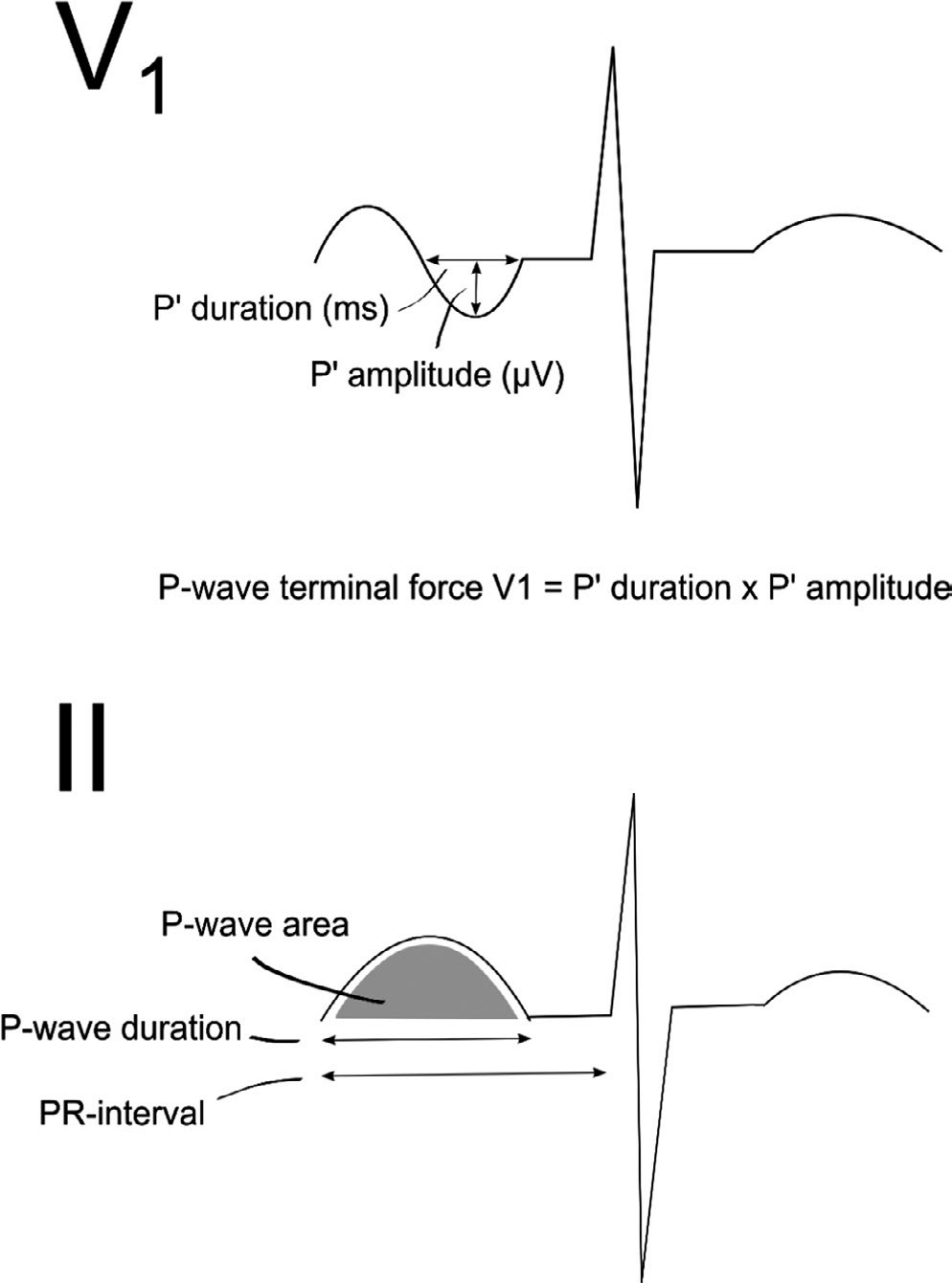
P‐wave measurements in lead V_1_ and II. P‐wave indices were measured digitally, manually in leads V_1_ and II. P‐wave terminal force was measured in lead V_1_ as the duration multiplied by the amplitude of the negative part of the P wave (µV ms). P‐wave area (µV ms), P‐wave duration (ms), and PR‐interval (ms) were measured in lead II

P‐wave terminal force was defined as the duration multiplied by the amplitude of the negative part of the P wave (P’_DURATION_ · P’_AMPLITUDE_), measured in µV ms in lead V_1_. For the analysis, the remaining PWIs were taken from measurements in lead II, as this lead provides a good view of the P wave (Meek & Morris, 2002). All P waves in lead II were positive. P_DURATION_ was measured from the on‐set of the P‐wave deflection to its return to the isoelectric line, measured in milliseconds. P_AREA_ was measured as the area under the curve of the positive deflection of the P wave (Figure 2). P_AREA/DURATION_ index was calculated as the ratio of P_AREA_ and P_DURATION_.

The inter‐rater variability was assessed by 2 raters using 20 randomly selected ECGs. One rater was used to assess intrarater variability, measuring the same 20 ECGs twice. In addition, inter‐rater variability was compared with automated measures.

### 2.5 Statistics

Continuous variables are presented as median and interquartile range (IQR) and compared with Kruskal‐Wallis tests. Categorical variables are presented as frequency (percentage) and compared with chi‐square tests. Two‐tailed tests of significance are reported and the level of statistical significant was set at 5%. The reproducibility was assessed for intra‐, inter and manual‐automatic variability using interclass correlation coefficient (ICC).

The Kaplan‐Meier method was used to calculate the absolute risk of all‐cause mortality and the Aalen‐Johansen method to calculate the absolute risk of AF. The association between PWI and the AF‐hazard rate was analyzed by cause‐specific Cox regression. To allow that extremely low and extremely high values of a biomarker both can be associated with increased AF‐hazard rate, we categorized the markers P_AREA/DURATION_ index and P_AREA_ according to their 20% and 80% quintiles. Reported were two AF‐hazard ratios and all‐cause mortality hazard ratios with 95% confidence limits (CI) using the middle 60% of the marker values as reference. All analyses with AF as endpoint were adjusted for Framingham score variables (Schnabel et al., 2009). All‐cause mortality was adjusted for age, sex, smoking, diabetes mellitus, and systolic blood pressure. To graphically illustrate the relation between PWI and AF‐hazard rate, we also applied restricted cubic splines with three knots (Harrell, 2001). For prediction analysis area under the ROC curve (AUC), we used the categorized P‐wave parameters. P_DURATION_ and PTF were categorized by known cut off points from literature (P_DURATION_ > 120 ms and PTF > 4,000 µV ms) (Magnani et al., 2010).

R Core Team (2018) and StataCorp (2013) were used for statistical analysis.

## 3 RESULTS

The study included 632 participants with no apparent heart disease at baseline. Table 1 shows the baseline characteristics for the overall study population and for three groups of the P_AREA/DURATION_ index in lead II, divided into the lowest quintile, the three middle quintiles joined (20%–80%) and the highest quintile of the index. P_AREA/DURATION_ index in the lower quintiles was associated with lower heart rate, and with smaller other PWIs in general. Apart from these differences, the groups were comparable in relation to other known risk factors of AF. Median follow‐up time was 14.7 (14.5;14.9) years. During this time, there were 68 cases of incident AF and 233 deaths.

**Table 1:** Baseline characteristics of study participants and comparison of characteristics in groups of lowest quintile, Q20‐Q80 and highest quintile of P‐wave area/P‐wave duration index in lead II

Variable	Level	Lowest quintile (*n* = 127)	Q20‐Q80 (*n* = 378)	Highest quintile (*n* = 127)	Total (*n* = 632)	p‐value
Gender	Male	77 (60.6)	220 (58.2)	68 (53.5)	365 (57.8)	.50
Age	55	35 (27.6)	73 (19.3)	20 (15.7)	128 (20.3)	
	60	28 (22.0)	88 (23.3)	30 (23.6)	146 (23.1)	
	65	18 (14.2)	83 (22.0)	33 (26.0)	134 (21.2)	
	70	23 (18.1)	76 (20.1)	24 (18.9)	123 (19.5)	
	75	23 (18.1)	58 (15.3)	20 (15.7)	101 (16.0)	.27
BMI	Underweight	1 (0.8)	6 (1.6)	5 (3.9)	12 (1.9)	
	Normal	32 (25.2)	125 (33.1)	55 (43.3)	212 (33.5)	
	Overweight	63 (49.6)	177 (46.8)	50 (39.4)	290 (45.9)	
	Obese	31 (24.4)	70 (18.5)	17 (13.4)	118 (18.7)	.01
Smoker	Yes	62 (48.8)	171 (45.2)	62 (48.8)	295 (46.7)	.68
Systolic Blood pressure, mmHg		155 [30] (120, 220)	150 [30] (100, 235)	160 [40] (100, 250)	155 [30] (100, 250)	.20
Heart rate, beats pr. min		66 [12] (48, 95)	70.5 [15.8] (48, 107)	78 [15.5] (49, 112)	71 [16] (48, 112)	<.0001
Systolic murmur	Grade 0	86 (67.7)	252 (66.7)	84 (66.1)	422 (66.8)	
	Grade 1–2	36 (28.3)	119 (31.5)	40 (31.5)	195 (30.9)	
	Grade 3–4	5 (3.9)	7 (1.9)	3 (2.4)	15 (2.4)	.87
Diabetes	Present	13 (10.2)	44 (11.6)	13 (10.2)	70 (11.1)	.86
Hypertension treatment	Present	34 (26.8)	105 (27.8)	39 (30.7)	178 (28.2)	.76
Cholesterol		6 [1.3] (2.5, 8.4)	6 [1.4] (3.3, 11.0)	6.1 [1.4] (1.9, 8.4)	6 [1.3] (1.9, 11.0)	.52
PR‐interval		163.3 [30] (121.7, 226.7)	165 [25] (115, 275)	163.3 [20.8] (123.3, 240.0)	165 [25] (115, 275)	.83
P‐wave area, lead II (µV·ms)		4,781 [1688] (1,063, 11,656)	8,438 [2422] (4,094, 14,063)	11,969 [2531] (7,844, 17,313)	8,453 [4143] (1,063, 17,313)	<.001
P‐wave duration, lead II (ms)		91 [26] (35, 128)	106 [18] (63, 149)	106 [12] (56, 143)	104 [18] (35, 149)	<.001
P‐wave terminal force, lead V_1_ (µV·ms)		2,798 [1939] (506, 8,779)	3,918 [2334] (737, 11,559)	5,840 [4064] (670, 18,926)	3,937 [2743] (506, 18,926)	<.001

Note

Values are *n* (%) or median interquartile range [IQR] (range).

### 3.1 Association analyses

**Figure 3:**
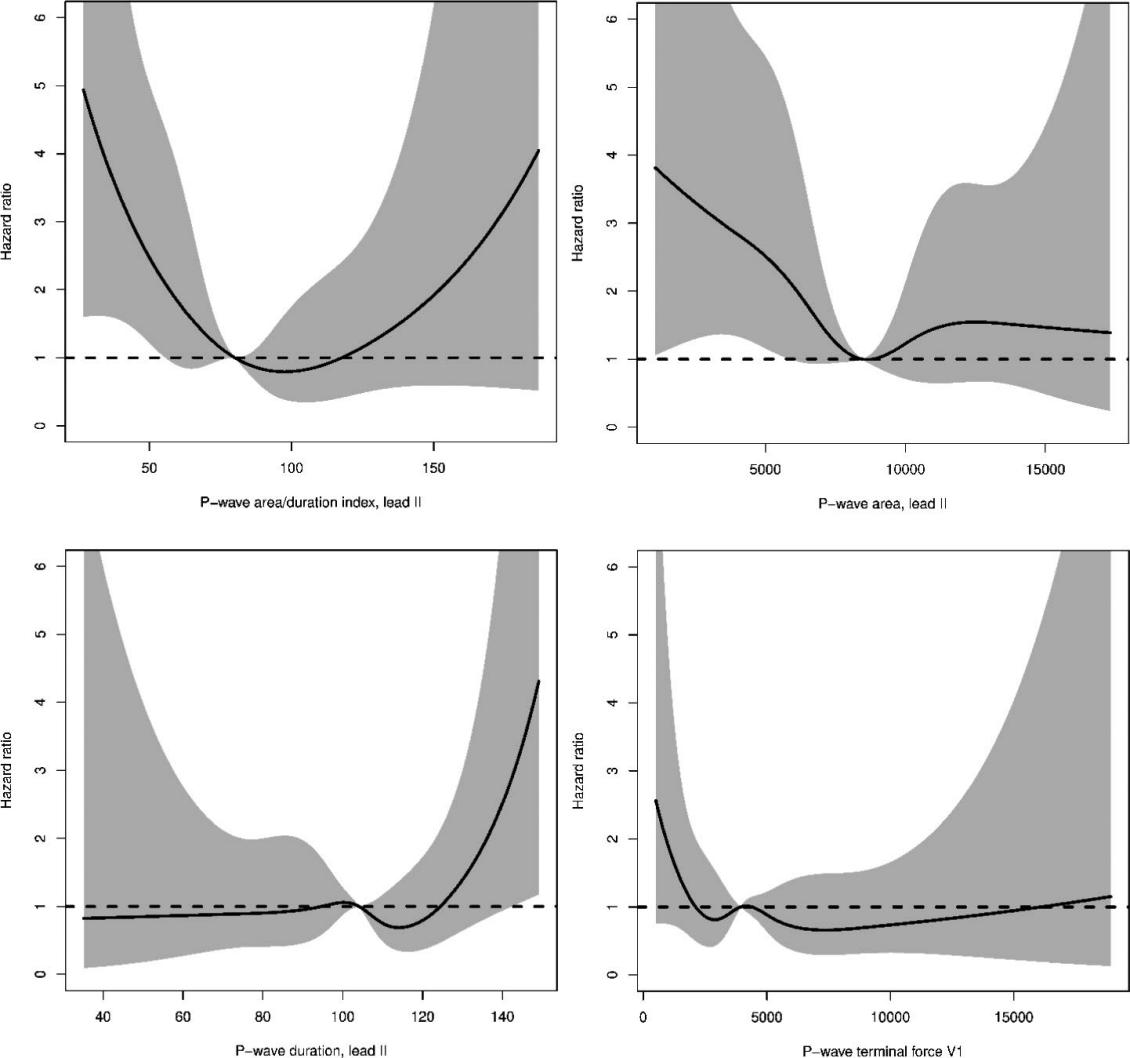
Restricted cubic splines of Hazard rate for atrial fibrillation (AF) and P‐wave indices. Restricted cubic spline analysis of the P‐wave area/P‐wave duration index and other PWI in relation to the hazard ratio of AF

*AF:* P_AREA/DURATION_ index and P_AREA_ were both associated with increased rate of AF in their lowest quintiles (Table 2). P_DURATION_ and PTF were not associated with rate of AF in these analyses. Adding heart rate to the cox regression models did not alter the results. Restricted cubic spline analyses showed a U‐shaped association between P_AREA/DURATION_ and risk of AF. However, the highest quintile did not reach statistical significance (Figure 3).

**Table 2:** Association of P‐wave indices with adjusted hazard rates for AF and mortality

P‐wave indices	AF (HR)	95% CI	*p*‐value	Mortality (HR)	95% CI	*p*‐value
P‐wave area/P‐wave duration index, lead II (Lowest quintile^a^)	2.80	(1.64,4.79)	<.001	1.17	(0.84,1.63)	.36
P‐wave area/P‐wave duration index, lead II (highest quintile^a^)	1.25	(0.64,2.46)	.52	1.39	(1.01,1.92)	.04
P‐wave area, lead II (Lowest quintile^a^)	2.16	(1.25,3.75)	.006	1.03	(0.73,1.44)	.88
P‐wave area, lead II (highest quintile^a^)	1.03	(0.54,1.97)	.93	1.06	(0.76,1.47)	.75
P‐wave duration, lead II above 120 ms	1.81	(0.95,3.45)	.07	1.03	(0.68,1.56)	.88
P‐wave terminal force V_1_ above 4,000	0.86	(0.52,1.41)	.55	1.03	(0.79,1.34)	.85

Note

Atrial fibrillation (AF) adjusted for Framingham AF risk factors: age, gender, systolic blood pressure, body mass index, PR‐interval, and systolic murmur. Mortality adjusted for age, gender, smoking, systolic blood pressure, cholesterol, and diabetes.

a Compared with quintile 2–4 as references.

*Mortality:* P_AREA/DURATION_ index in its highest quintile was associated with mortality (Table 2). None of the other PWI were associated with mortality.

### 3.2 Discrimination

The addition of P_AREA/DURATION_ index significantly improved AF risk discrimination after 15 years of follow‐up. Receiver Operating Characteristic analyses (AUC) for the Framingham score variables, different PWI, and addition of PWI into the Framingham model are shown in Table 3.

**Table 3:** Difference in area under the ROC curve (AUC) in relation to atrial fibrillation with Framingham score variables alone versus addition of P‐wave indices for 5 and 15 years of follow‐up

P‐wave indices	Years since electrocardiography	AUC (CI)	Delta AUC (CI)	*p*
Framingham variables alone	5	78.0 [70.2;85.8]		
P‐wave area/P‐wave duration index, lead II	5	78.6 [70.4;86.9]	0.6 [−8.2;9.4]	.89
P‐wave area, lead II	5	78.1 [70.4;85.8]	0.1 [−7.4;7.6]	.98
P‐wave duration, lead II	5	77.6 [68.1;87.1]	−0.4 [−3.5;2.7]	.80
P‐wave terminal force, lead V_1_	5	77.7 [69.4;86.0]	−0.3 [−2.4;1.8]	.78
Framingham variables alone	15	53.5 [29.1;77.9]		
P‐wave area/P‐wave duration index, lead II	15	62.0 [33.6;90.3]	8.5 [0.6;16.3]	.034
P‐wave area, lead II	15	59.1 [32.3;85.9]	5.6 [−0.6;11.8]	.076
P‐wave duration, lead II	15	54.8 [29.7;79.8]	1.3 [−1.7;4.3]	.41
P‐wave terminal force, lead V_1_	15	54.4 [29.6;79.2]	0.9 [−0.9;2.8]	.33

Note

Area under the ROC curve for P‐wave area/P‐wave duration index and other PWI when added to Framingham score variables.

### 3.3 Absolute risk

**Figure 4:**
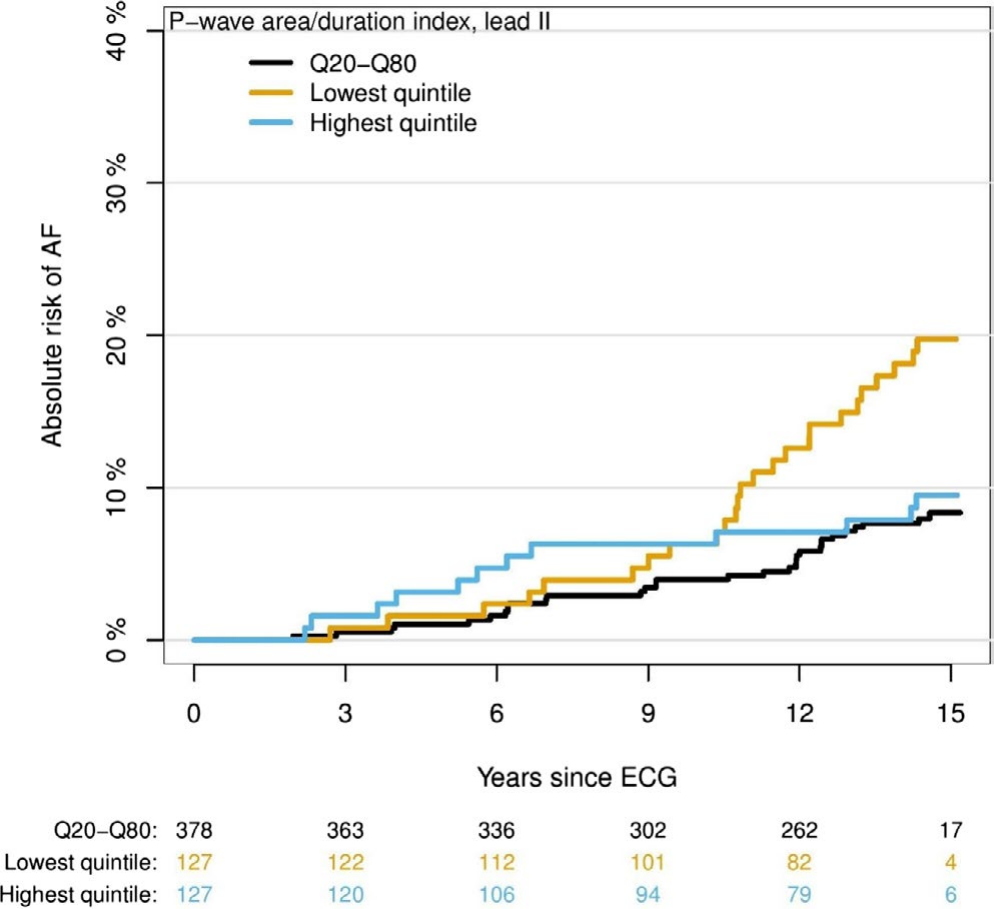
Absolute risk of atrial fibrillation (AF) by P‐wave area/P‐wave duration index in three groups. Absolute risk of AF in years since baseline electrocardiography divided into lowest, highest, and middle quintiles for the P_AREA/DURATION_ index

**Figure 5:**
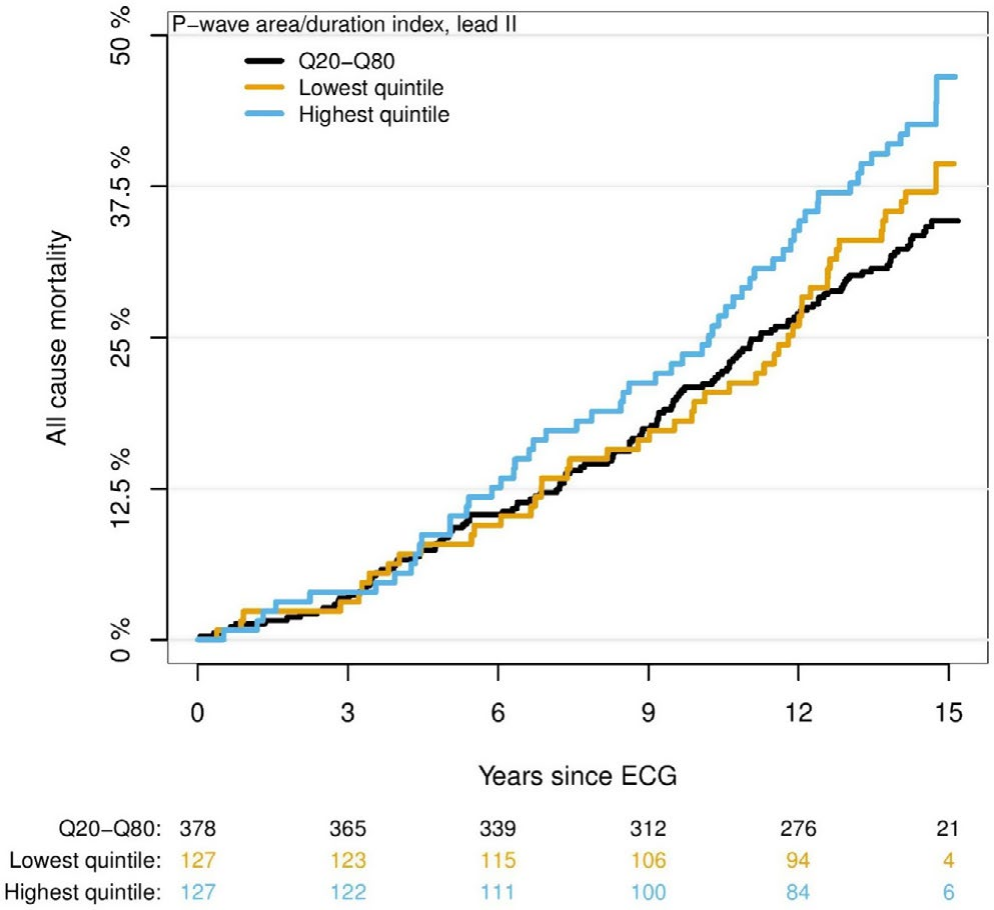
Absolute risk of mortality for P‐wave area/P‐wave duration index. Absolute risk of mortality in years since baseline electrocardiography divided into lowest, highest, and middle quintiles for the P_AREA/DURATION_ index

Figure 4 shows Aalen‐Johansen curves for the events of AF and Figure 5 show Kaplan‐Meier survival curves for the events of all‐cause mortality for different P_AREA/DURATION_ categories.

### 3.4 Inter‐ and intrarater variability for P‐wave measurement

The intraobserver reliability for P_DURATION_, P_AREA_, and PTF had ICC values of .96, .99, and .99, respectively. The interobserver reliability for two observers for P_DURATION_, P_AREA_, and PTF had ICC values of .94, .96, and .97, respectively. We also investigated the variability between manual and automated ECG analysis, with ICC values for P_DURATION_ and PTF of 0.89 and 0.88, respectively.

## 4 DISCUSSION

This was an exploratory study that aimed to assess the prognostic value of the P_AREA/DURATION_ index and other PWI in incidence of AF The major finding of this study is that a flat P wave (Figure 1) expressed as a small P_AREA/DURATION_ index in lead II, is significantly associated with long‐term incidence of AF after relevant adjustments. Furthermore, it was shown that the P_AREA/DURATION_ index improved the Framingham risk model for AF in this population. An increased P_AREA/DURATION_ index was associated with all‐cause mortality.

Many cardiovascular diseases and concomitant conditions increase the risk of developing AF (Kirchhof et al., 2016). Cumulative exposure to risk factor induces a progressive structural remodeling of the atria (Kirchhof et al., 2016). The hallmark of structural atrial remodeling in the progressive process of AF is atrial fibrosis and dilatation (Kirchhof et al., 2016). Fibrosis seems to be an important underlying cause of AF because of conduction heterogeneity, triggered activity, and reentry (Burstein & Nattel, 2008). Histologically, the atrial tissue in patients with AF is shown to be more fibrotic compared to atrial tissue in patients without AF (Burstein & Nattel, 2008). Increased fibrotic tissue, loss of muscle, and dilatation of the atria are correlated to more frequent and less reversible incidents of AF (Burstein et al., 2008). Prolonged total atrial activation time is an indirect marker of atrial fibrosis (Gorenek et al., 2017). Fibrosis is known to decrease conduction (Nguyen, Qu, & Weiss, 2014), thereby giving a smaller and longer signal on the ECG (Michelucci et al., 2002). A small P_AREA/DURATION_ index may, therefore, be considered a possible marker for both atrial dilatation and fibrosis. These changes can prolong LA's activation time and thus be considered as the equivalent of left bundle branch block and poor R‐wave progression in the ventricles. These changes may be due to increased pressure or volume load on atrium, for example, due to hypertension or valvular disease or a slow degeneration due to genetic factors or environmental factors.

It is noteworthy that the effect of P_AREA/DURATION_ on the risk of AF could be demonstrated only after several years of follow‐up. Thus, a smaller P_AREA/DURATION_ may represent initial atrial changes that after years will lead to AF. Another possibility is that many of these subjects have had undetected paroxysmal AF for years before it was ultimately incident.

The association of P_AREA/DURATION_ in its highest quintile and increased rate of mortality is also notable and may be due to dilatation and hypertrophy of atrium due to other diseases with increased work load on the atria, such as pulmonary artery hypertension, severe hypertension, or similar.

Our findings are consistent with previous research that has demonstrated a significant association between a small P_AREA_ and rate of AF (Magnani et al., 2015). However, P_AREA_ has not been widely investigated. To our knowledge, only two other studies have examined P_AREA_ in relation to AF risk. The ARIC study showed that both large and small maximum P_AREA_ were significantly associated with AF (Magnani et al., 2015). The Framingham study showed no increased risk associated with maximum P_AREA_ (Magnani et al., 2015). In fact, the Framingham study found no increased AF risk associated with any PWIs. Other studies have shown that prolonged P_DURATION_ is associated with AF (Magnani et al., 2011), and one study has found that shorter P_DURATION_ (Nielsen et al., 2015) is also significantly associated with AF. The associations for P_AREA_ and P_DURATION_ in relation to AF risk can therefore possibly also be considered as U‐shaped curves, where both smaller and larger than normal measures are signs of atrial dysfunction and thereby possible predictors of AF. This is also clear from restricted cubic spline curves in this study, showing a U‐shaped trend for the P_AREA/DURATION_ index, however, with a broad confidence interval in the high end, possibly due to too few observations.

In contrast to previous studies, however, we found no association between PTF and rate of AF. PTF is particularly sensitive to electrode placement (Rasmussen et al., 2019). Therefore, the lack of association in this study may in part be due to the occurrence of high placement of the precordial electrodes in clinical settings (García‐Niebla, 2009; McCann, Holdgate, Mahammad, & Waddington, 2007; Medani, Hensey, Caples, & Owens, 2017; Rajaganeshan, Ludlam, Francis, Parasramka, & Sutton, 2008), and the impact that high placement has on measurements. High placement of V_1_‐V_2_ electrodes increases the negative component of the P wave in V_1_ and V_2_ (García‐Niebla, 2009; Ishikawa & Yanagisawa, 1981). In this study, other PWI has been measured in lead II. We argue that the limb leads are most reliable for measuring PWI. Lead II is known to be the best lead for evaluating P waves (Meek et al., 2002).

### 4.1 Limitations

Our study sample was comprised mostly of Caucasians, so our results may not be transferrable to other ethnic groups. Furthermore, our sample consisted of subjects over 55 years of age, most with at least one cardiovascular risk factor, so our results may not be transferrable to a younger, healthy population. Additionally, there is a possibility that not all cases of AF were recorded, because of the paroxysmal nature of this arrhythmia.

In this study, automated analysis and quantification were not performed; however, we have demonstrated that PWI can be measured manually with high intra and inter‐rater reproducibility that correlate well with automated measurements.

### 4.2 Clinical perspective

AF prediction using simple, clinically available tools that enable clinicians to identify subjects with increased risk of developing AF would be of high value in pre‐emptive strategies of AF‐related complications. Our study is consistent with previous studies that suggest PWI could be a potential candidate, and we demonstrated that the new index (P_AREA/DURATION_) is a strong marker with additive effect on Framingham AF score and an association with all‐cause mortality. However, further studies are necessary in order to establish the reliability of this new index as a marker for AF prediction. As P_AREA/DURATION_ also reflects aspects of P‐wave morphology, it makes it easier for clinicians to visualize and capture P‐wave abnormalities.

## 5 CONCLUSION

A flat P wave as expressed by a small P_AREA/DURATION_ index in lead II, possibly as an ECG marker of atrial muscle loss and prolongation of conduction time, is associated with increased rate of incident AF beyond known AF risk factors.

## CONFLICT OF INTEREST

None.

## AUTHORS’ CONTRIBUTION

A.S. and M.U.R. conceived the study. M.U.R and A.F. performed the ECG measurements. J.K. assisted in validating the measurement method. All statistical analysis was done by T.A.G.. P.K., A.F., B.L., J.K., H.D., U.D. participated in discussions and contributed to the design and implementation of the research, to the analysis of the results and to the writing of the manuscript. All authors reviewed and commented on the manuscript.

## ETHICAL APPROVAL

The Copenhagen Holter Study was approved by the Regional Ethics Committee of Copenhagen and Frederiksberg. The study complies with the Helsinki Declaration. All participants provided their written informed consent.
